# Accurate FDG PET tumor segmentation using the peritumoral halo layer method: a study in patients with esophageal squamous cell carcinoma

**DOI:** 10.1186/s40644-018-0169-1

**Published:** 2018-09-26

**Authors:** Sungmin Jun, Jung Gu Park, Youngduk Seo

**Affiliations:** 1 Department of Nuclear Medicine, Kosin University Gospel Hospital, Kosin University College of Medicine, Busan, 49297 South Korea; 2 Department of Radiology, Kosin University Gospel Hospital, Kosin University College of Medicine, Busan, 49297 South Korea; 3 Department of Nuclear Medicine, Busan Seongso Hospital, Suyeong-ro, Nam-gu, Busan, 48453 Republic of Korea

**Keywords:** Peritumoral halo layer, Tumor segmentation, FDG PET/CT, Esophageal cancer, Reliability test, Tumor length

## Abstract

**Background:**

In a previous study, FDG PET tumor segmentation (SegPHL) using the peritumoral halo layer (PHL) was more reliable than fixed threshold methods in patients with thyroid cancer. We performed this study to validate the reliability and accuracy of the PHL method in patients with esophageal squamous cell carcinomas (ESCCs), which can be larger and more heterogeneous than thyroid cancers.

**Methods:**

A total of 121 ESCC patients (FDG avid = 85 (70.2%); FDG non-avid = 36 (29.8%)) were enrolled in this study. In FDG avid ESCCs, metabolic tumor length (ML) using SegPHL (ML_PHL_), fixed SUV 2.5 threshold (ML_2.5_), and fixed 40% of maximum SUV (SUVmax) (ML_40%_) were measured. Regression and Bland-Altman analyses were performed to evaluate associations between ML, endoscopic tumor length (EL), and pathologic tumor length (PL). A comparison test was performed to evaluate the absolute difference between ML and PL. Correlation with tumor threshold determined by the PHL method (PHL tumor threshold) and SUVmax was evaluated.

**Results:**

ML_PHL_, ML_2.5_, and ML_40%_ correlated well with EL (R^2^ = 0.6464, 0.5789, 0.3321, respectively; *p* < 0.001) and PL (R^2^ = 0.8778, 0.8365, 0.6266, respectively; p < 0.001). However, ML_2.5_ and ML_40%_ showed significant proportional error with regard to PL; there was no significant error between ML_PHL_ and PL. ML_PHL_ showed the smallest standard deviation on Bland-Altman analyses. The absolute differences between ML and PL were significantly smaller for ML_PHL_ and ML_40%_ than for ML_2.5_ (*p* < 0.0001). The PHL tumor threshold showed an inverse correlation with SUVmax (σ = − 0.923, p < 0.0001).

**Conclusions:**

SegPHL was more accurate than fixed threshold methods in ESCC. The PHL tumor threshold was adjusted according to SUVmax of ESCC.

## Introduction

F-18 fluorodeoxyglucose (FDG) PET/CT is a noninvasive modality for staging and localization of various malignant tumors that have high glucose metabolism. FDG PET/CT can provide a variety of metabolic parameters, such as maximum standardized uptake value (SUVmax), metabolic tumor volume (MTV) [1, 2], total lesion glycolysis (TLG) [3], and metabolic tumor length (ML) [4, 5]. Among these, SUVmax is the most commonly used FDG PET parameter because the measurement is easy and operator independent. Although SUVmax is a widely used parameter, it is a representation of the highest metabolic value of only one pixel within a metabolically active tumor. Thus, the total burden of the primary tumor cannot be evaluated by SUVmax.

MTV and TLG can provide volumetric metabolic information on malignant tumors. MTV is a measurement of the volume of a tumor with a high metabolism, while TLG is defined as the product of mean SUV and MTV [6]. A variety of methods have been used to measure and segment MTV. These include visual segmentation methods (SegVisual), where the region of interest is drawn manually [7, 8], fixed SUV threshold methods (SegSUV) [3, 6], fixed percentage of SUVmax threshold methods (Seg%) [3, 6], adaptive threshold methods (SegAdaptive) [6, 9], and gradient methods (SegGradient) [6, 10, 11]. Among these various segmentation approaches, SegSUV or Seg% is widely used to measure MTV because nearly all PET/CT workstations have an auto-segmentation program for fixed threshold methods. Accurate segmentation of MTV is also important for accurate measurement of TLG. Because TLG is a product of MTV and mean SUV (SUVmean), accurate MTV segmentation is essential for reliable TLG measurement. Furthermore, an accurate SUVmean estimate also requires accurate MTV segmentation. If segmented MTV is largely different from the true pathologic tumor volume, TLG will be inaccurate. ML is the length of the tumor on the PET image. The threshold determination for ML is similar to that for MTV [12].

SegAdaptive and SegGradient are known to be more accurate than SegSUV or Seg% [6, 10, 11]. The main principle of SegAdaptive is to adapt the percentage threshold of the phantom or tumor according to signal-to-background ratio [6, 9, 13]. Several optimal regression functions can be obtained by fitting various regression models [6, 9, 13, 14]. MTV using SegGradient is a method used to detect a large gradient change in radioactivity around the tumor. The large gradient change is located in the outmost peripheral portion of the tumor, and its location is used to define the tumor margin [11, 15, 16]. However, a specialized program and workstation, PET Edge® (MIM Software Inc., Cleveland, OH, USA), is needed to determine MTV using SegGradient.

Jun et al. [17] recently introduced a new method for MTV segmentation. They found a distinct layer between the tumor and background activity using a 10-step color scale with specific window level settings and named the distinct layer the “peritumoral halo layer (PHL).” Segmentation using the PHL method (SegPHL) was more reliable than MTV segmented by SegSUV or Seg%. Although SegPHL might be reliable, the previous study was performed in patients with a small and visually homogeneous type of tumor, namely papillary thyroid carcinoma [17]. Thus, further validation of large, heterogeneous tumors is needed to determine if SegPHL can be widely applied in clinical settings.

Our aim was to compare variously segmented MLs (i.e., ML segmented by SUV 2.5 (ML_2.5_), 40% of SUVmax (ML_40%_), and PHL (ML_PHL_)) of esophageal squamous cell carcinoma (ESCC) with pathological tumor length (PL) and to identify whether SegPHL could be used to reliably define tumor margin in ESCC, which can be large and/or heterogeneous.

## Methods

### Subjects

**Fig. 1:**
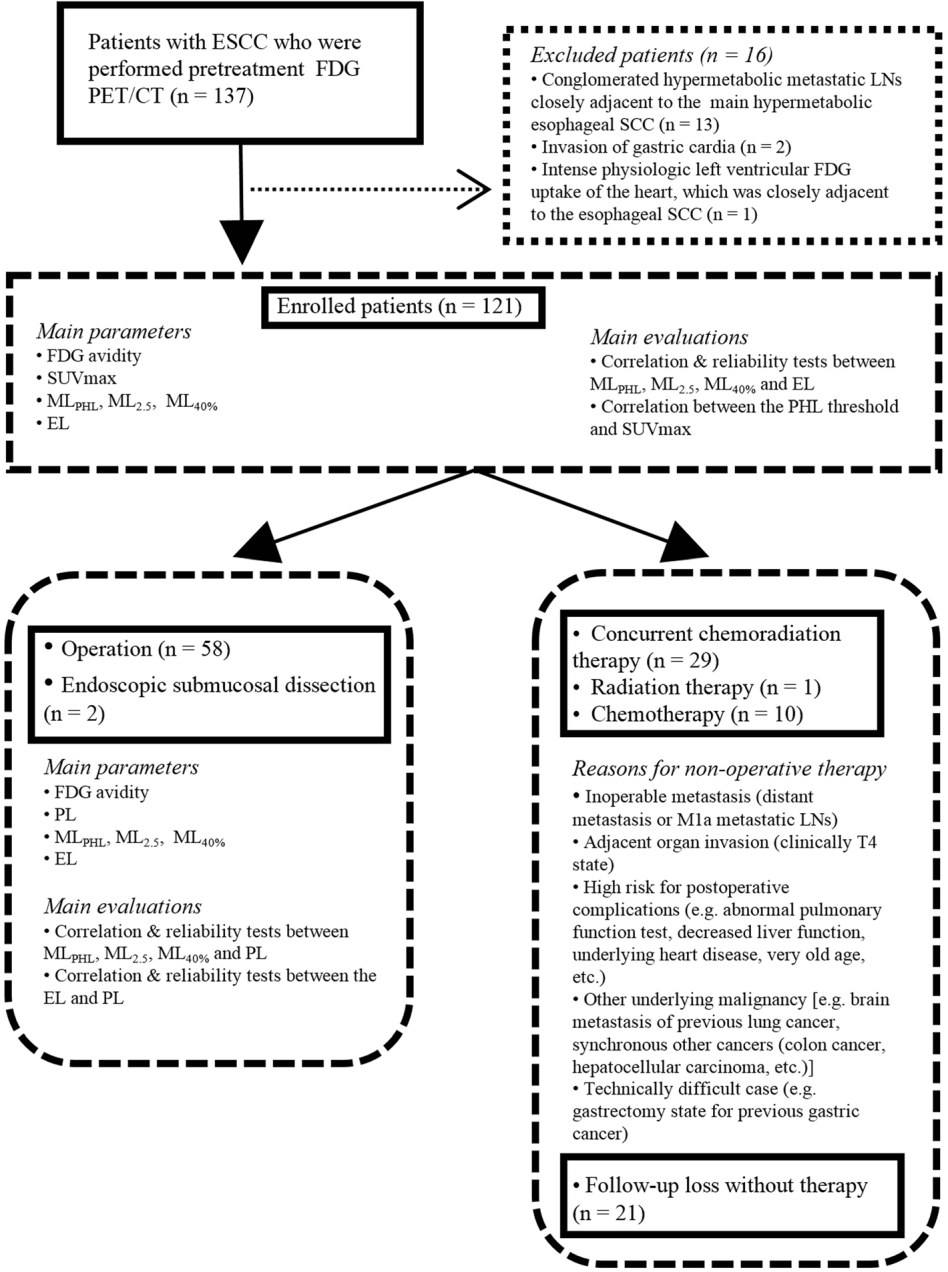
Flowchart of patient selection, treatment methods, main parameters, and evaluations

This retrospective study was approved by the institutional review board of our hospital and performed in accordance with the Helsinki Declaration. Between January 2013 and June 2017, 137 consecutive patients who had undergone pretreatment FDG PET/CT were evaluated for this study. Patient selection, main parameters, and evaluations are represented graphically in Fig. 1. Among the 137 patients, 16 were excluded for the following reasons: (1) conglomerated hypermetabolic metastatic lymph nodes (LNs) closely adjacent to the main hypermetabolic ESCC (*n* = 13, 2) invasion of the gastric cardia (*n* = 2), and intense physiologic left ventricular FDG uptake of the heart that was closely adjacent to the ESCC (*n* = 1). A total of 121 patients were finally enrolled in this study.

Among the 121 patients, 58 underwent esophagectomy, 2 underwent endoscopic submucosal dissection (ESD), 29 underwent concurrent chemoradiation therapy (CCRT), 1 underwent radiation therapy, 10 underwent chemotherapy only, and 21 were lost to follow-up. Reasons for non-operative therapy are illustrated in Fig. 1. Of the total patients, 111 (91.7%) were men, and 10 (8.3%) were women. Tumor locations were the upper third of the esophagus in 18 (14.9%), mid third in 64 (52.9%), lower third in 31 (25.6%), upper and mid esophagus in 6 (5.0%), and mid and lower esophagus in 2 (1.7%). The median age of the 121 patients at the time of FDG PET/CT pretreatment was 63 years (range: 33–85 years). The time interval between FDG PET/CT and esophagectomy or ESD was less than 6 weeks (median = 7.5 days; range = 1–39 days).

### Measurement of endoscopic length

Among the 121 enrolled patients, 116 underwent evaluation of esophagogastroduodenoscopy (EGD) at our hospital (Table 1). Endoscopic length (EL) was routinely measured and reported by the operator in centimeters. The distance from the incisors was recorded on the basis of markings on the endoscope shaft at the distal edge of the ESCC. Thereafter, the endoscope was drawn to the proximal edge of the ESCC. The distance from the incisors was again estimated. EL was defined as the difference in the distances of the proximal and distal edges from the incisors [18].

**Table 1:** Comparison of demographics and characteristics of primary esophageal SCCs according to FDG avidity on PET/CT (*n* = 121)

	FDG-avid (*n* = 85)	FDG-non-avid (*n* = 36)	*P* value
Age (years)			0.5403
Median, range	63, 48–85	67, 33–81	
Sex (male/female)	79/6	32/4	0.4818
Tumor location			0.3251
Upper third	14	4	
Mid third	43	21	
Lower third	20	11	
Upper to mid	6	0	
Mid to lower	2	0	
Pretreatment endoscopy in our hospital (performed/not performed)	83 / 2	33 / 3	0.1553
Treatment methods			0.0405*
Operation	37	22	
Endoscopic submucosal dissection	0	1	
Concurrent chemoradiation therapy	25	4	
Radiation therapy alone	1	0	
Chemotherapy alone	9	1	
Follow-up loss without any therapy (follow-up loss/treatment in our hospital)	13 / 72	8 / 28	0.4321

*Statistically significant

### Acquisition of FDG PET/CT

Patients fasted for at least six hours before FDG injection (370–444 MBq). Serum glucose level was measured before FDG injection. Scanning was performed 50–70 min after injection of FDG using a Biograph Duo PET/CT scanner (Siemens Healthcare, Erlangen, Germany) or a Biograph 16 PET/CT scanner (Siemens Healthcare, Erlangen, Germany). Patients were asked to rest before acquisition of PET/CT images. Before the PET scan, a low-dose CT scan (5 mm slices) at an interval of 5 mm was performed for attenuation correction and anatomic co-registration. Intravenous contrast material was not used. PET images were acquired using an acquisition time of 3 min per table position, with an approximate 28% overlap. Images were obtained from the skull base to the proximal thigh with the patient in supine position. Images were reconstructed by a 3D row-action maximum-likelihood algorithm for iterative and ordered-subset expectation maximization using the Biograph system. PET images were corrected for attenuation using a CT-derived transmission map. Voxel size after reconstruction was 2.65 × 2.65 × 2.65 mm.

### Identification of PHL and determination of tumor threshold on FDG PET/CT

All FDG PET/CT studies were reviewed on a workstation (Siemens Syngo.via, Siemens Healthcare, Erlangen, Germany). First, we evaluated FDG avidity of the primary ESCC. We considered ESCC to be FDG-avid if abnormal focal or ellipsoid tumoral hypermetabolic activity was visible in the esophagus. If there was no abnormal FDG-avid lesion, we recorded the case as FDG-non-avid ESCC. We measured the SUVmax of FDG-avid ESCCs.

The PHL was identified, and the tumor threshold (PHL tumor threshold) was determined as described by Jun and colleagues for papillary thyroid cancer [17]. ESCCs, however, can be visually heterogeneous, unlike papillary thyroid carcinomas, which are almost always small and visually homogeneous. Therefore, the visual tumor pattern was also described in this section.

**Fig. 2:**
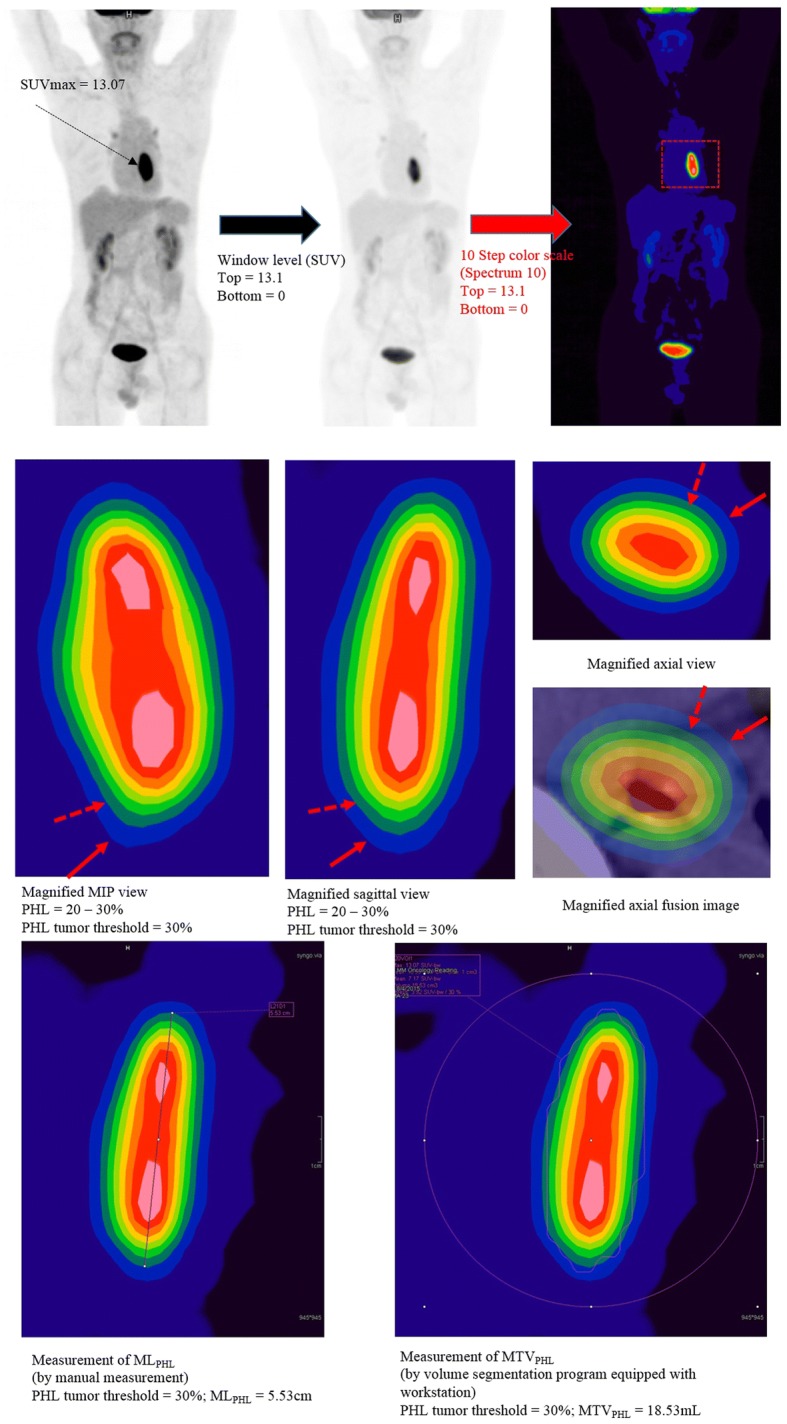
Representative images to determine PHL tumor threshold and to measure ML_PHL_ and MTV_PHL_. Step 1 (upper): PHL image settings. A segmental hypermetabolic lesion (SUVmax = 13.07) is identified in the mid esophagus (upper left). The window level is set in SUV units. The top value is set to slightly higher than SUVmax (top value 13.1 > 13.07), and the bottom value is set to zero (upper middle). Thereafter, the color scale is changed to a 10-step color scale, which was Spectrum 10 in our study (upper right). A magnified view is used to determine background activity and PHL. Magnification of the ESCC (red dotted box) is performed. Step 2 (middle): Magnified view of the ESCC using PHL image settings (red dotted box in the upper right image). In this image, each layer represents 10% of SUVmax. The hottest core is > 90% of SUVmax. There are two hot cores in this ESCC. PHL is located between the tumor and the background activity. In this image, the background activity is the dark blue layer (10–20% of SUVmax), and the PHL is located at 20–30% of SUVmax (light blue layer; red arrows). Therefore, the PHL tumor threshold is 30% of SUVmax (i.e., the innermost portion of the PHL; red dotted arrow). Layer thickness increases abruptly in the PHL. Step 3 (lower): Measurement of ML_PHL_ and MTV_PHL_. After we determined PHL tumor threshold, we manually measured ML_PHL_ using the ruler tool of the workstation (lower left). MTV_PHL_ was measured by the auto-segmentation program after applying PHL tumor threshold (lower right; 30% of SUVmax in this ESCC)

An example of identification of PHL and PHL tumor threshold is provided in Fig. 2. PET window level and color scale for PHL identification (PHL image settings) were set as follows:

1. PET contrast window level was set in SUV units (not Bq/mL or % units);

2. The top value of the window level was set slightly higher than the SUVmax of the ESCC (e.g., if the SUVmax was 13.00 ~ 13.09, the top value was set to 13.1), and the bottom value was set to 0;

3. Color scale was changed to a 10-step color scale. We used the Spectrum 10 scale of our workstation.

After inputting these settings, the PHL and PHL tumor threshold were identified according to the following steps:

1. The view around the ESCC was magnified for accurate identification of the PHL.

2. The PHL was identified by an abrupt increase in layer thickness with minimal or mild distortion of the main tumor contour and was between tumoral uptake and background activity [17]. In the present study, the PHL was easy to determine by examining magnified MIP images.

3. The PHL tumor threshold was determined using PHL. Using the PHL imaging settings described above, each color layer represents 10% of the SUVmax. PHL was located between the tumor and the background. Thus, the outermost point of the tumor was the same as the innermost point of the PHL. If the PHL was located at 20–30% of SUVmax, the percentage SUVmax in the innermost portion of the PHL (i.e., PHL tumor threshold) was determined to be 30% (Fig. 2).

**Fig. 3:**
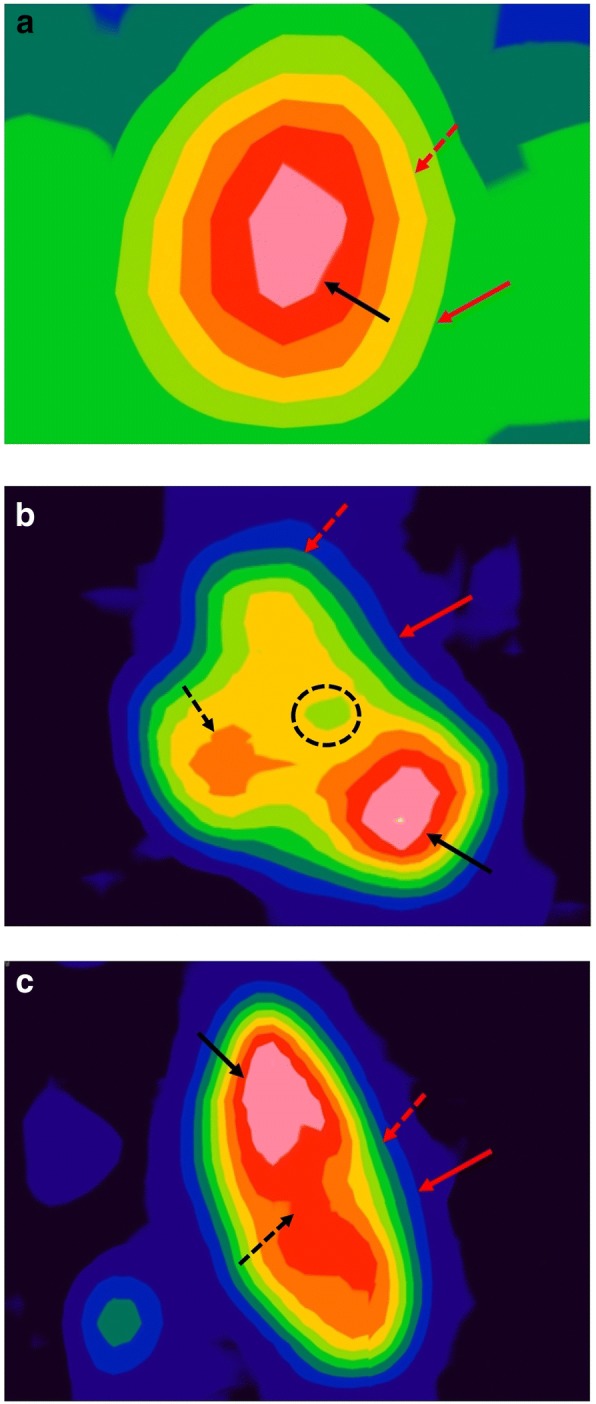
Visual patterns of the tumors. **a** Visually homogeneous pattern. The hottest core (black arrow) was located in the center of the ESCC. There were no significant irregular layers in the tumor. PHL (50–60% of SUVmax; yellowish green layer) was located between the tumor and the background activity (red arrow). PHL tumor threshold (red dotted arrow) was determined to be 60% of SUVmax (i.e., the innermost portion of the PHL). **b** Visually heterogeneous pattern. The hottest core was eccentrically located in the ESCC, and a small cold defect was visible (small yellowish green portion in black dotted circle). An irregular layer was also present (black dotted arrow). PHL (20–30% of SUVmax; light blue layer) was located between the tumor and the background activity (red arrow). PHL tumor threshold (red dotted arrow) was determined to be 30% of SUVmax (i.e., the innermost portion of the PHL). **c** Visually heterogeneous pattern. The hottest core was eccentrically located in the ESCC (black arrow). A prominent irregular layer was present (black dotted arrow). PHL (20–30% of SUVmax; light blue layer) was located between the tumor and the background activity (red arrow). PHL tumor threshold (red dotted arrow) was determined to be 30% of SUVmax (i.e., the innermost portion of the PHL)

After this series of steps, tumoral uptake patterns were classified as visually homogeneous or visually heterogeneous [19]. Tumoral uptake of the ESCC comprised the hottest cores and regular or irregular tumor layers (not the PHL) (Fig. 3). In visually homogeneous ESCCs, the hottest core was located in the center of the ESCC and was surrounded by regular tumor layers (Fig. 3a). In contrast, in visually heterogeneous ESCCs, the hottest core or the hottest multiple cores were located in the eccentric portion of the tumor. In these heterogeneous tumors, several irregular inner layers were visible around the eccentric hottest core, and single or multiple regular outer layers of the tumor enveloped the hottest core and irregular inner layers (Figs. 3b, c). Regardless of visual tumor heterogeneity, the PHL was always located between tumoral uptake and background activity. Classification of visual tumor heterogeneity in our study was similar to that used by Tixier et al. [19] with the exception of the PHL image settings.

The locations of the PHL and visual tumor heterogeneity were determined by two nuclear medicine physicians who were blinded to pathologic tumor length (PL) and EL values. If the two physicians disagreed, the final decision was made by consensus.

### Measurements of metabolic tumor length

After determination of PHL tumor threshold, we measured ML_PHL_, ML_2.5_, and ML_40%_ of the ESCC. A straight line was drawn using the ruler tool of our workstation in sagittal view (Fig. 2). ML_PHL_ and ML_40%_ were measured using the PHL tumor threshold and fixed at 40% of SUVmax on the PHL image setting. ML_2.5_ was measured on SUV2.5 iso-contour images with a PET window bottom value of 2.50 SUV and a top value of 2.51 SUV. When SUVmax of the tumor was lower than 2.5, ML_2.5_ was recorded as 0.

### Evaluation of pathologic tumor length (pathologic reference standard)

PL measurements of ESCCs are performed routinely in our hospital. PL was measured by the following procedure. An incision was made along the longitudinal esophageal axis. Thereafter, the esophageal specimen with a straight alignment was set on a flat table. The specimen was sent for pathological examination after preservation in 10% neutral buffered formalin. The PL of the ESCC specimen was measured using a ruler and recorded.

### Statistical analyses

Major parameters and analyses in our study are presented in Fig. 1. Demographic and tumor data according to FDG avidity of ESCC were compared using Fisher’s exact test, chi-square test, or Mann-Whitney U test. Weighted Kappa statistics were performed to evaluate inter-observer agreement between the two nuclear medicine physicians for PHL tumor threshold. Regression analyses were performed to evaluate the correlations between ML_PHL_, ML_2.5_, ML_40%_, EL, and PL. Bland-Altman plots and Pearson correlation coefficients were used to evaluate the reliability of ML, EL, and PL. Relationships between PHL tumor threshold and SUVmax were evaluated by Spearman’s rank correlation. MedCalc® for Windows (MedCalc Software, Mariakerke, Belgium) was the statistical software package used for all statistical analyses.

## Results

### Clinical situation according to FDG avidity of ESCC

A total of 121 ESCC patients were enrolled in our study. Demographics and other characteristics of ESCCs according to FDG avidity are shown in Tables 1 and 2. There were 85 FDG avid ESCCs (visually homogeneous: *n* = 48; visually heterogeneous: *n* = 37) and 36 FDG non-avid ESCCs. FDG avidity of ESCCs was related to inoperability (*p* = 0.0405), EL (*p* = 0.0042), PL (*p* = 0.0001), presence of metastatic LNs on FDG PET/CT (*p* = 0.0005), presence of M1a metastatic LN or distant metastasis (*p* = 0.0037), and pathologic depth of invasion (*p* < 0.0001). The frequency of metastatic LNs on pathologic specimens tended to be higher in FDG avid ESCCs than in FDG non-avid ESCCs but without statistical significance (*p* = 0.0941). Age, sex, and tumor location did not differ according to FDG avidity of ESCC.

**Table 2:** Comparison of endoscopic length, pathologic length, and frequency of metastasis according to FDG avidity of the primary ESCC

	FDG-avid (*n* = 85)	FDG-non-avid (*n* = 36)	*P* value
Endoscopic passage to distal portion of the tumor* (possible/impossible)	81/2	33/0	1.0000
Endoscopic length (cm)**			0.0042^†^
Median, range	3.5, 1.0–9.0	2.0, 0.5–9.0	
Pathologic length (cm)***			0.0001^†^
Median, range	3.5, 0.9–7.6	1.7, 0.15–6.0	
Metastatic LN on FDG PET/CT (yes/no)	40 / 45	5 / 31	0.0005^†^
M1a metastatic LN or distant metastasis on FDG PET/CT (yes/no)	21 / 64	1 / 35	0.0037^†^
Presence of metastatic LN on pathologic specimens**** (yes/no)	18 / 19	5 / 16	0.0941
Pathologic depth of invasion (T stage)***			< 0.0001^†^
Basement membrane (Tis)	0	3	
Mucosa (T1a)	2	9	
Submucosa (T1b)	11	11	
Muscularis propria (T2)	9	0	
Adventitia (T3)	15	0	
Invasion of adjacent structures (T4)	0	0	

*Pretreatment endoscopic evaluation was performed in 116 patients at our hospital

**Measurement of endoscopic length was impossible in 2 patients with total esophageal obstruction

***Measurement of pathologic length and depth of invasion was possible for 60 patients who were treated by surgery (*n* = 58) or ESD (*n* = 2). There was no T4 case in these patients

****Frequency test of pathologically confirmed metastatic LNs was performed for 58 operative patients

LN = lymph node; ESD = endoscopic submucosal dissection

^†^Statistically significant

### Inter-observer agreement for PHL determination

Tumor segmentation by PHL was available for all FDG-avid ESCCs (*n* = 85). Inter-observer agreement for PHL tumor threshold was very good (k = 0.936) (Table 3).

**Table 3:** Inter-observer agreement test for determination of PHL tumor threshold

	Observer A								
	PHL tumor threshold	20%	30%	40%	50%	60%	70%	80%	90%
Observer B	20%	2							
	30%	1	40						
	40%		2	13					
	50%			2	6				
	60%				1	2	2		
	70%						5	1	
	80%						1	5	
	90%								2

k = 0.936

### Correlations between ML and EL in FDG-avid ESCCs and reliability testing

In 121 study patients, pretreatment EGD was performed in 116. Among the 116 patients, EL was measured in 114; measurements were impossible in two patients because of esophageal obstruction (Table 2). The median EL in the 114 patients was 3.0 cm (range 0.5–9.0 cm; interquartile range 2.0–5.0 cm). Among the 85 patients with FDG avid ESCC, EGD was performed for 83 (Table 1). EL was measured in 81 of those patients, while EL measurement was impossible in 2 patients because the endoscope could not pass through the ESCC. Therefore, MLs (ML_PHL_, ML_2.5_, and ML_40%_) and EL were compared in 81 patients. The median EL was 3.5 cm in FDG avid ESCCs (range 1.0–9.0 cm) (Table 2). Medians of ML_PHL_, ML_2.5_, and ML_40%_ were 3.81 cm (range 0.80–9.80 cm), 4.80 cm (range 0.00–10.30 cm), and 4.40 cm (range 1.88–9.39 cm), respectively. In the 85 FDG avid ESCCs, there was only 1 case where the SUVmax of the ESCC was lower than 2.5 and the ML_2.5_ was zero.

**Fig. 4:**
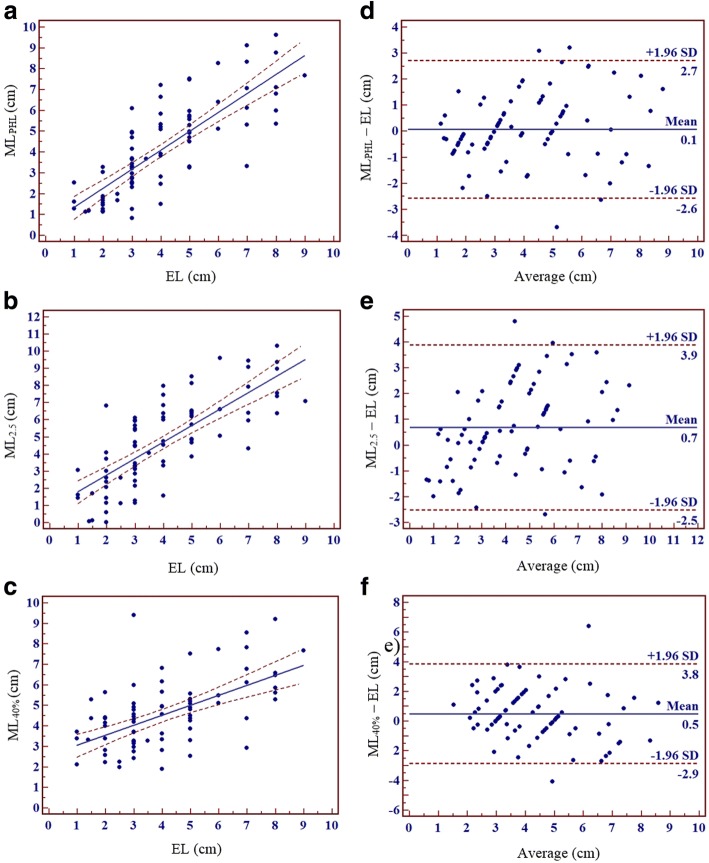
Regression analyses and Bland-Altman plots of ML_PHL_, ML_2.5_, and ML_40%_ versus EL. ML_PHL_, ML_2.5_, and ML_40%_ were significantly correlated with EL (**a**: R^2^ = 0.6464, *p* < 0.001; **b**: R^2^ = 0.5789, *p* < 0.001; **c**: R^2^ = 0.3321, *p* < 0.001). Bias and standard deviation were smallest between ML_PHL_ and EL (**d**–**f**)

ML_PHL_, ML_2.5_, and ML_40%_ were significantly correlated with EL (ML_PHL_: *p* < 0.001, slope = 0.91, R^2^ = 0.6464; ML_2.5_: p < 0.001, slope = 0.96, R^2^ = 0.5789; ML_40%_: *p* < 0.001, slope = 0.49, R^2^ = 0.3321) (Figs. 4a-c). In Bland-Altman analyses (Fig. 4d-f), the biases between ML_PHL_, ML_2.5_, and ML_40%_ and EL were 0.1 cm (limits of agreement = − 2.62 ~ 2.69 cm; SD = 1.3532), 0.7 cm (limits of agreement = − 2.53 ~ 3.89 cm; SD = 1.6357), and 0.5 cm (limits of agreement = − 2.87 ~ 3.83; SD = 1.7096), respectively. Bias and standard deviation were smallest for ML_PHL_ and EL. There was significant proportional error between ML_2.5_ and EL on the Bland-Altman plot (*r* = 0.3458, *p* = 0.0016). No significant error was found between ML_PHL_ or ML_40%_ and EL on the Bland-Altman plots (ML_PHL_: *r* = 0.2102, *p* = 0.0597; ML_40%_: *r* = − 0.2027, *p* = 0.0695).

### Comparisons between MLs, EL, and PL

**Fig. 5:**
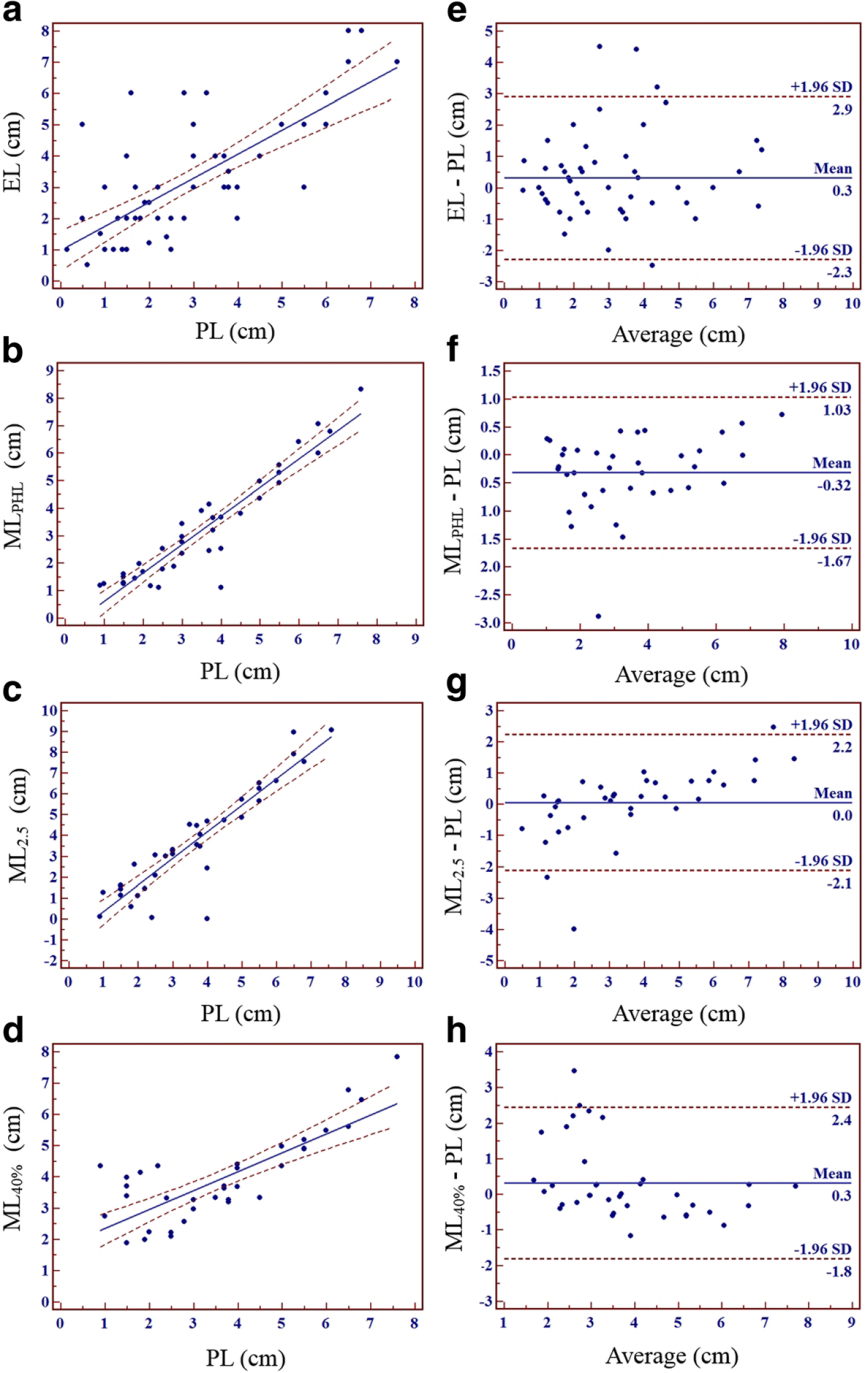
Regression analyses and Bland-Altman plots of EL, ML_PHL_, ML_2.5_, and ML_40%_ versus PL. EL, ML_PHL_, ML_2.5_, and ML_40%_ were significantly correlated with PL (**a**: R^2^ = 0.5482, *p* < 0.001; **b**: R^2^ = 0.8778, *p* < 0.001; **c**: R^2^ = 8365, *p* < 0.001; **d**: R^2^ = 0.6266, *p* < 0.001). The standard deviation was smallest between ML_PHL_ and PL (**d**–**f**). There was significant proportional error between ML_2.5_ or ML_40%_ and PL. However, no significant proportional error was found between ML_PHL_ and PL

Of the 121 study patients, PL measurements were performed in 60 (58 esophagectomy and 2 ESD patients) (Fig. 1). Among these 60 patients, 2 did not undergo EGD in our hospital. Correlation between EL and PL was assessed in 58 patients (56 esophagectomy and 2 ESD patients). EL was significantly correlated with PL (R^2^ = 0.5482, slope = 0.77, *p* < 0.001) (Fig. 5a). In Bland-Altman analysis (Fig. 5e) of the 58 patients, the bias between EL and PL was 0.3 cm (limits of agreement = − 2.29 ~ 2.90 cm, SD = 1.3254 cm). There was no significant proportional error between EL and PL on the Bland-Altman plot (*r* = 0.0629, *p* = 0.6390).

Of the 85 patients with FDG avid ESCCs, esophagectomy was performed in 37 (Table 1). ML and PL were compared in these 37 patients. ML_PHL_, ML_2.5_, and ML_40%_ were significantly correlated with PL (ML_PHL_: R^2^ = 0.8778, slope = 1.03, *p* < 0.001; ML_2.5_: R^2^ = 0.8365, slope = 1.27, *p* < 0.001; ML_40%_: R^2^ = 0.6266, slope = 0.61, *p* < 0.001) (Figs. 5b-d). In Bland-Altman analyses (Fig. 5f-h), the biases between ML_PHL_, ML_2.5_, and ML_40%_ and PL were − 0.32 cm (limits of agreement = − 1.67 ~ 1.03 cm; SD = 0.6869 cm), 0.0 cm (limits of agreement = − 2.13 ~ 2.23 cm; SD = 1.1117 cm), and 0.3 cm (limits of agreement = − 1.81 ~ 2.44 cm; SD = 1.0847 cm), respectively. The biases of ML_PHL_, ML_2.5_, and ML_40%_ were less than 0.5 cm. However, ML_PHL_ showed the smallest SD in Bland-Altman analysis.

There was no significant proportional error between ML_PHL_ and PL (*r* = 0.2902, *p* = 0.0815). However, there were significant proportional errors between ML_2.5_, ML_40%_, and PL on Bland-Altman plots (ML_2.5_: *r* = 0.6036, *p* = 0.0001; ML_40%_: *r* = − 0.4049, *p* = 0.0129). The proportional error of ML_2.5_ implied that the value of ML_2.5_ was smaller when average length was small and/or larger when average length was large. In contrast, the proportional error implied that ML_40%_ was larger when average length was small and/or smaller when average length was large.

**Fig. 6:**
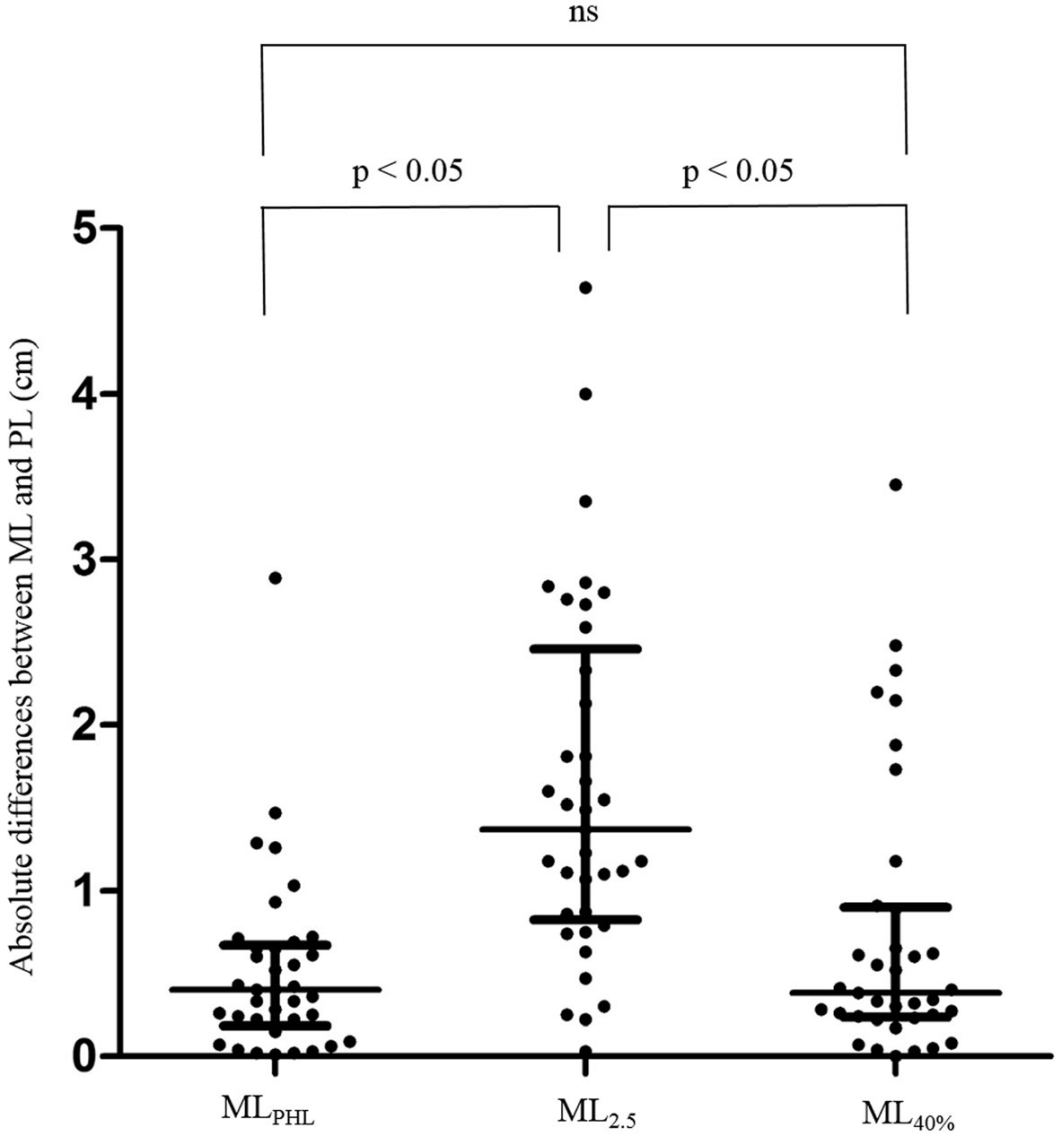
Absolute differences between MLs and PL. Each dot represents an absolute difference between ML and PL. The middle horizontal bars represent medians, and the vertical bars represent interquartile ranges. Absolute differences between PL and ML were significantly smaller for ML_PHL_ and ML_40%_ than for ML_2.5_ (Kruskal-Wallis test: *p* < 0.0001, *p* < 0.05 by Dunn’s post-hoc test)

The absolute differences between MLs (i.e., ML_PHL_, ML_2.5_, and ML_40%_) and PL were calculated as absolute value of (MLs - PL). The absolute difference was significantly smaller for ML_PHL_ and ML_40%_ than for ML_2.5_ (ML_PHL_: median 0.40 cm, interquartile range 0.20 ~ 0.66 cm; ML_2.5_: median 1.37 cm, interquartile range 0.84 ~ 2.40 cm; ML_40%_: median 0.38 cm. interquartile range 0.24 ~ 0.90; Kruskal-Wallis test: *p* < 0.0001) (Fig. 6).

### Effects of SUVmax on ML measurement

We evaluated the differences between MLs (i.e., ML_PHL_, ML_2.5_, ML_40%_) and PL according to SUVmax of ESCC. The difference between ML_PHL_ and PL was only weakly correlated with SUVmax (Spearman correlation: σ = 0.372, *p* = 0.0254). However, the difference between ML_2.5_ and PL showed a strong positive correlation with SUVmax (Spearman correlation: σ = 0.806, *p* < 0.0001). In contrast to ML_2.5_, the difference between ML_40%_ and PL was strongly negatively correlated with SUVmax (Spearman correlation: σ = − 0.789, *p* < 0.0001). These results suggested that ML_PHL_ was less affected by SUVmax of ESCC than were ML_2.5_ and ML_40%_.

### Changes in PHL tumor threshold according to SUVmax

**Fig. 7:**
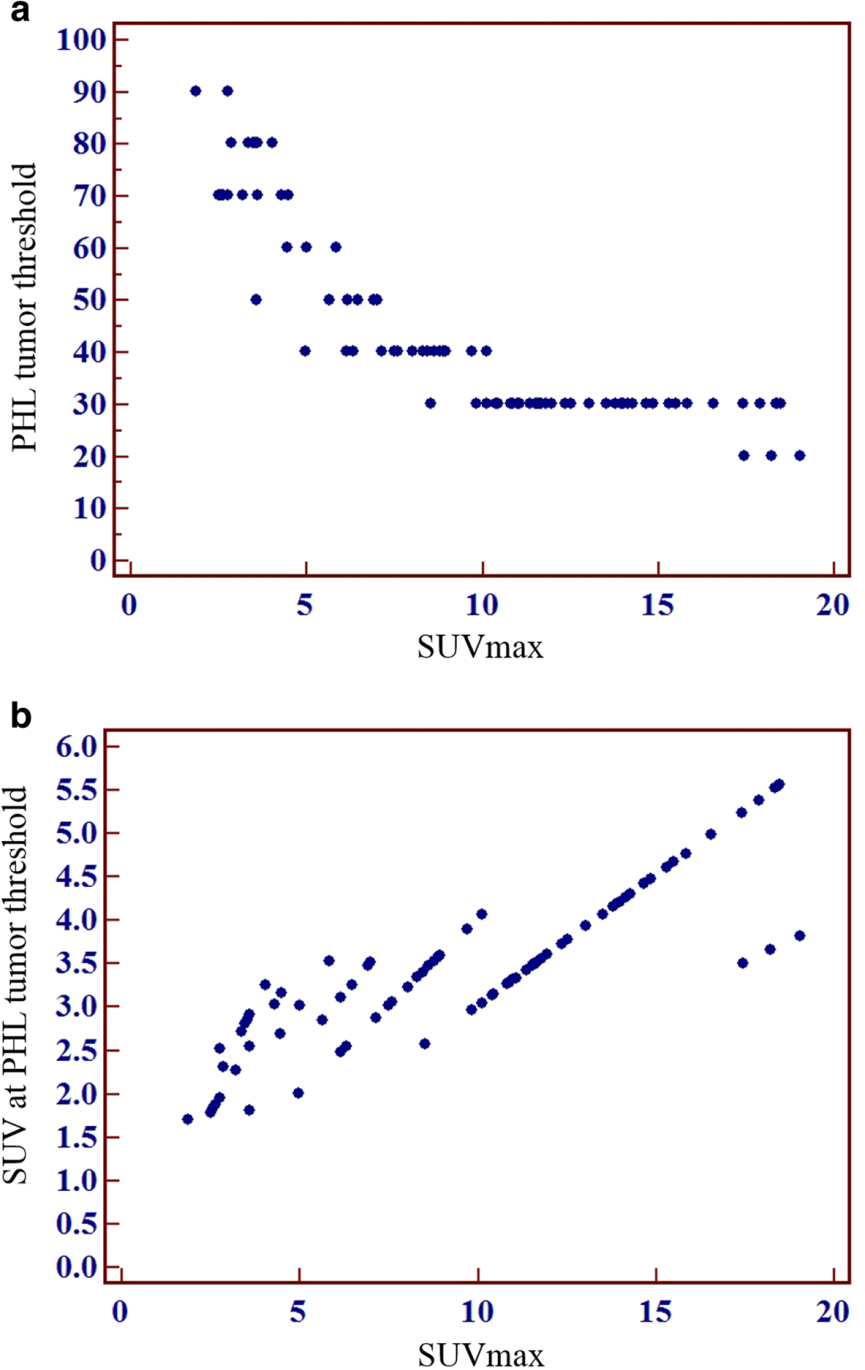
Relationship between SUVmax and PHL tumor threshold. PHL tumor threshold (% of SUVmax) showed a strong inverse correlation with SUVmax (**a**: σ = − 0.923, *p* < 0.0001). The SUV of PHL tumor threshold showed a strong positive correlation with SUVmax (**b**: σ = 0.891, *p* < 0.0001)

We evaluated the relationship between PHL tumor threshold and SUVmax in 85 FDG avid ESCCs as described in a previous study [17]. PHL tumor threshold (i.e., % SUVmax determined by the PHL method) showed a strong inverse correlation with SUVmax (σ = − 0.923, p < 0.0001; Fig. 7a). SUV at the PHL tumor threshold (i.e., SUVmax × PHL tumor threshold) showed a strong positive correlation with SUVmax (σ = 0.891, p < 0.0001; Fig. 7b).

## Discussion

The major finding of the current study is that SegPHL is more accurate and reliable for defining the tumor margin of ESCCs than are fixed threshold methods. In a previous study, Jun et al. [17] reported that SegPHL was more reliable than fixed threshold methods for delineation of the margins of thyroid cancers. However, the thyroid cancers examined in that study were very small and visually homogeneous. They reported that PHL abruptly increased in layer thickness and was located between the tumor and the background. In the present study, we demonstrated that SegPHL was also accurate in ESCCs, which can be large and/or visually heterogeneous. ML_PHL_ was highly correlated with PL (Fig. 5), the bias and standard deviation of ML_PHL_ were small on the Bland-Altman plot (Fig. 5), and there were no serious proportional errors visible on the plot. The absolute difference between ML_PHL_ and PL was smaller than that of ML_2.5_ and PL (Fig. 6). ML_PHL_ was less affected by SUVmax of the ESCC than were ML_2.5_ and ML_40%_. The PHL tumor threshold (% of SUVmax) showed an inverse correlation with SUVmax (Fig. 7). The characteristics of the PHL were similar to those reported in a previous study [17]; PHL also showed an abrupt increase in layer thickness and was located between the tumor activity and the background. Furthermore, PHL tumor threshold changed according to SUVmax of the ESCC (Fig. 7), consistent with a previous PHL study [17].

The most widely used segmentation methods are SegSUV and Seg%. However, accurate tumor segmentation with a fixed threshold is impossible with these methods because the optimal tumor threshold changes according to SUVmax or signal-to-background ratio [13, 20, 21]. The volume of a tumor with faint FDG uptake is highly overestimated by Seg%. In addition, MTV using SegSUV underestimates the MTV of a tumor with low metabolic activity and overestimates that of a tumor with high metabolic activity [6]. These shortcomings of Seg% and SegSUV were also found in our present study. The difference between ML_2.5_ and PL was larger when SUVmax was higher, and the difference between ML_40%_ and PL was larger when SUVmax was lower. However, ML_PHL_ was less affected by SUVmax. The results of our study suggest that the PHL method could accurately segment FDG avid tumor regardless of SUVmax.

SegVisual has been reported to be more accurate than the fixed threshold method in some cases [7]. Although accurate tumor segmentation may be possible with visual tumor delineation, the SegVisual has several limitations. First, the reproducibility of SegVisual is low [22]. Second, visual tumor perception can be altered by the PET window level setting and the color scale [23–25]. Third, the process is quite slow, because no auto-segmentation program is used.

The SegPHL could be considered as a SegVisual because it involves the visual location of the background and the PHL. However, there are several major differences between SegPHL and SegVisual. First, inter-observer agreement for the SegPHL is very good (Table 3), unlike with SegVisual. This good agreement may be due to the use of specific PHL image settings (i.e., PET window level: top = SUVmax, bottom = 0; color scale = 10-step color scale). Among our study population of 121 cases, PHL tumor threshold was concordant in 111 (91.7%). The second difference between SegPHL and SegVisual is that SegPHL uses commercial auto-segmentation programs to measure MTV_PHL_ with a known, accurate PHL tumor threshold (i.e., % of SUVmax). Use of an auto-segmentation program allows more rapid measurement of MTV than is possible with SegVisual.

SegPHL may also be similar to SegGradient. The unique characteristics of PHL are location between the tumor and the background and abrupt increase in layer thickness compared to the inner layer without significant distortion of tumor shape [17]. In the gradient method, the tumor margin is considered the point at which a large gradient change occurs in the PET/CT image [11]. A previous study suggested that PHL was very similar to the large gradient change of SegGradient [17]. We also hypothesized that the location of the PHL would likely be very similar to that of the large gradient change of SegGradient. Furthermore, like SegGradient [11], SegPHL was more accurate than fixed threshold methods. The main differences between SegPHL and SegGradient are as follows. First, PHL tumor threshold is determined by visual inspection of the tumor using PHL image settings. In contrast, SegGradient is determined using a special workstation. Second, an accurate percentage threshold can be determined by SegPHL (i.e., % of SUVmax). However, there is no way to determine an accurate % threshold of SegGradient. Third, although SegPHL is similar to SegGradient, the exact point at which there is a large gradient change cannot be determined using SegPHL. In our study, we defined PHL tumor threshold as the starting point of the PHL (i.e., the innermost point of the PHL), as in Jun et al. [17]. However, the large gradient change of SegGradient could be located in the outermost point or in the middle of the PHL. Another consideration is that the large gradient change of SegGradient might not be represented by a single % of SUVmax (i.e., the SUV of the large gradient change may differ in different parts of the tumor).

SegAdaptive is based on calibrated curves, where accurate % threshold changes as signal-to-background ratio changes [6, 13]. In this method, as the signal-to-background ratio increases, the accurate % threshold decreases [13]. Clinicians can choose an optimal regression equation to determine the accurate % threshold. Based on a phantom study, SegAdaptive is superior to fixed threshold methods [13, 26]. In our present study, PHL tumor threshold showed a strong inverse correlation with SUVmax of ESCC (σ = − 0.923, *p* < 0.0001; Fig. 7a), as reported for SegAdaptive in the phantom study. Similarly, Jun et al. [17] reported that PHL tumor threshold was inversely correlated with SUVmax of thyroid cancer.

Several studies have compared ML, MTV, PL, and pathologic tumor volume [12, 21, 27, 28]. The results of these reports suggest that there is no single optimal SUV or % of SUVmax threshold that can be used to define an accurate tumor margin [6]. In our present study, PHL tumor threshold (%) showed a significant inverse correlation with SUVmax of ESCC (σ = − 0.923, *p* < 0.0001; Fig. 7a), and SUV of PHL tumor threshold had a significant positive correlation with SUVmax of ESCC (σ = 0.891, p < 0.0001; Fig. 7b). Our results are consistent with previous studies that compared ML, MTV, PL, and pathologic tumor volume [12, 21, 27] and with a previous PHL study of thyroid cancer [17]. The number of cases where ML was compared with PL was similar between our present study and other previous studies (Zhong et al. [12]: *n* = 37 in esophageal cancer; Borakati et al. [28]: *n* = 21 in esophageal cancer).

An accurate tumor segmentation method should satisfy the following conditions. First, the segmented ML has to have a high degree of correlation with PL. Second, the bias and standard deviation should be small on Bland-Altman plots. Third, no serious proportional errors should be found in Bland-Altman analysis. Fourth, the segmented ML should not be highly affected by SUVmax of ESCC. Fifth, the segmented ML should correlate strongly with EL. Among ML_PHL_, ML_2.5_, and ML_40%_, only ML_PHL_ satisfied these conditions. Although ESCCs in our present study were definitely larger and more visually heterogeneous than the thyroid cancers examined in a previous PHL study [17], SegPHL was the most accurate segmentation method among SegPHL, SegSUV, and Seg%. Therefore, we conclude that SegPHL is far more accurate than fixed threshold methods.

Our study had several limitations. First, the study design was a retrospective analysis. Thus, there might have been operator-dependent bias in the measurement of EL and PL. Second, comparison of MTV and pathologic tumor volume was impossible because a vertical incision along the esophagectomy specimen had to be performed to evaluate pathologic depth of invasion. The vertical incision process changed the ellipsoid tumor shape of the ESCC into a planar shape. Because a change in tumor shape could introduce serious bias, we only assessed the correlation between ML and PL. Third, PL was measured after formalin fixation, which could shrink the esophagectomy specimen.

## Conclusion

In conclusion, SegPHL was more accurate than fixed threshold methods compared with PL. PHL tumor threshold was adjusted according to SUVmax of ESCC. We conclude that SegPHL might be used to accurately define the tumor margins of ESCCs.

## Abbreviations

CCRT: concurrent chemoradiation therapy
EGD: esophagogastroduodenoscopy
EL: endoscopic tumor length
ESCC: esophageal squamous cell carcinoma
ESD: endoscopic submucosal dissection
FDG: F-18 fluorodeoxyglucose
ML: metabolic tumor length
ML_2.5_: metabolic tumor length using SUV 2.5 threshold
ML_40%_: metabolic tumor length using fixed 40% of SUVmax threshold
ML_PHL_: metabolic tumor length using the peritumoral halo layer method
MTV: metabolic tumor volume
PHL: peritumoral halo layer
PL: pathologic tumor length
Seg%: segmentation using fixed % of SUVmax
SegAdaptive: segmentation using the adaptive threshold method
SegGradient: segmentation using the gradient method
SegPHL: segmentation using the PHL method
SegSUV: segmentation using fixed SUV threshold
SegVisual: segmentation using visual delineation
SUV: standardized uptake value
SUVmax: maximum standardized uptake value
TLG: total lesion glycolysis
